# Supplementary material for: Scientific literature on carbon dioxide removal revealed as much larger through AI-enhanced systematic mapping
Source: Nat Commun. 2025 Jul 18;16:6632. doi: 10.1038/s41467-025-61485-8 (PMC12274466; doi:10.1038/s41467-025-61485-8)
Supplement: Supplementary file 1 — Supplementary Information [file 41467_2025_61485_MOESM1_ESM.pdf]

# Supplementary Information of
# Scientific literature on carbon dioxide removal revealed as much larger through AI-enhanced systematic mapping

Sarah Lück , Max Callaghan, Malgorzata Borchers, Annette Cowie, Sabine Fuss, Matthew Gidden, Jens Hartmann, Claudia Kammann, David P. Keller, Florian Kraxner, William F. Lamb, Niall Mac Dowell, Finn Müller-Hansen, Gregory F. Nemet, Benedict S. Probst, Phil Renforth, Tim Repke, Wilfried Rickels, Ingrid Schulte, Pete Smith, Stephen M Smith, Daniela Thrän, Tiffany G. Troxler, Volker Sick, Mijndert van der Spek, Jan C. Minx

## Table of content


| | |
|---|---|
| Supplementary Figures 1-11 | 2 |
| Supplementary Tables 1-6 | 10 |
| Supplementary Method 1: Queries and Validation Dataset | 18 |
| Supplementary Method 2: Coding | 18 |
| Supplementary Method 3: Classification | 18 |
| Supplementary Method 4: Estimation of Confidence Interval for Absolute Counts | 19 |
| Supplementary Note 1: Coding Guideline including Supplementary Tables 7-11 | 21 |
| Supplementary References | 33 |

## Supplementary Figures

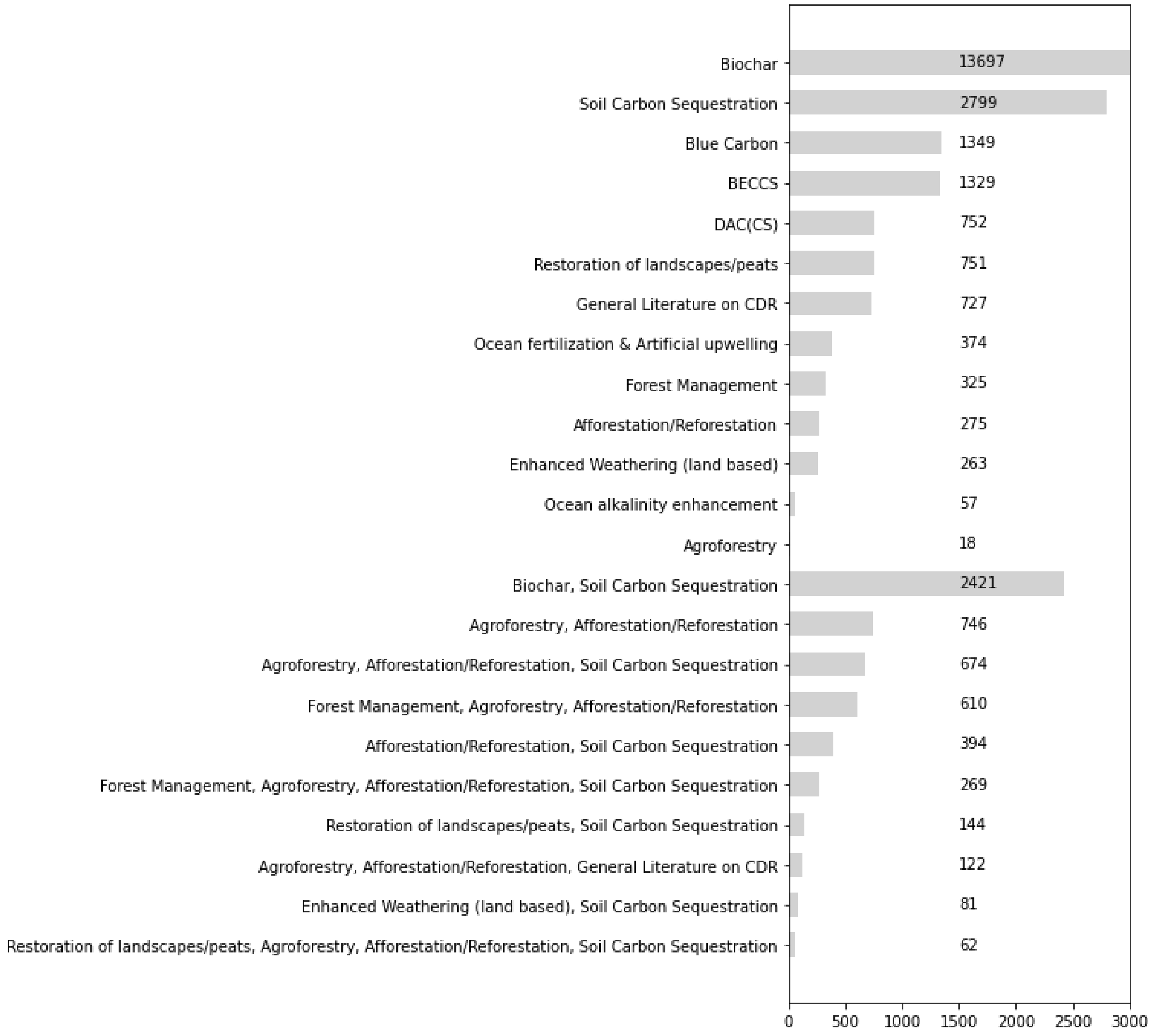


Supplementary Figure 1: Related to Figure 1. Summary of CDR option classifications across papers, indicating the number of categories with overlapping counts. Each paper is listed once. The first 13 rows show categories where a paper was assigned a single CDR option, while the following 10 rows present the most common double and triple categorizations. The first row is truncated, and all numbers shown are exact counts.

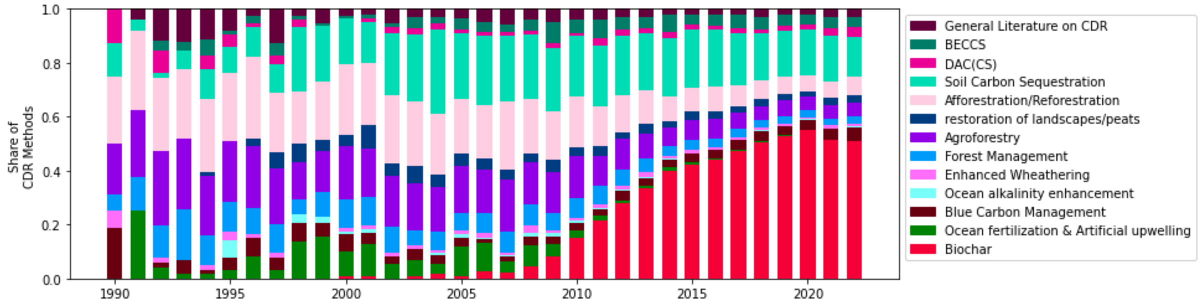


Supplementary Figure 2: Share of CDR option covered in scientific publications. Multiple options per publication are possible. A more complete list of all counts per option is published in the Supplementary Figure 1. Colorblind-friendly version of Figure 2 b

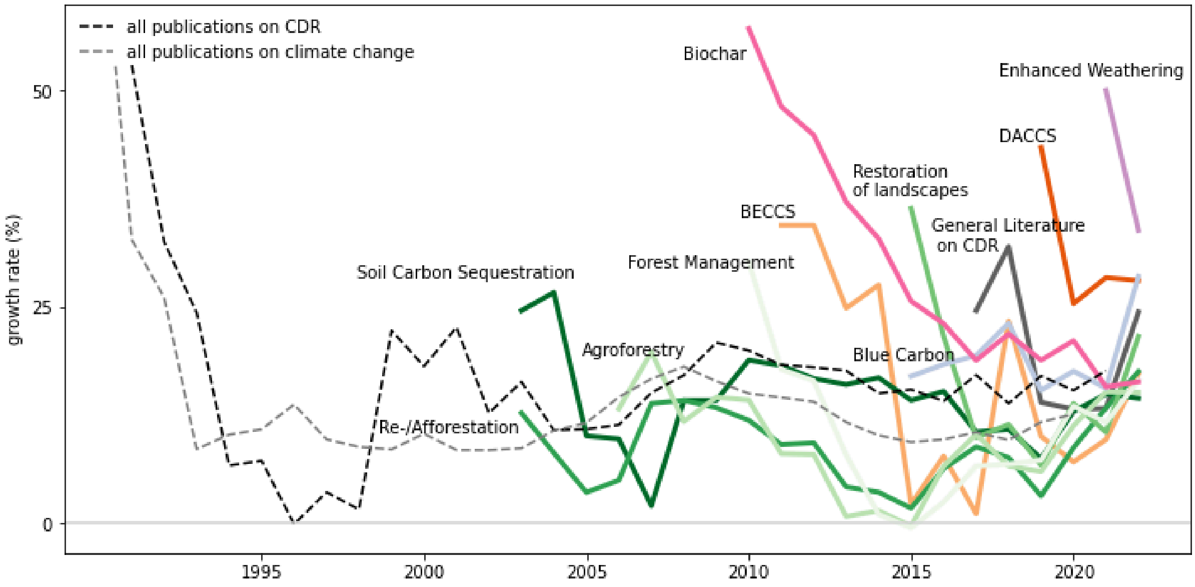


Supplementary Figure 3: Annual growth rate of the scientific literature on CDR, climate change and individual options. Growth rate is only calculated if there were more than 50 publications in total available. Colorblind-friendly version of Figure 2 c

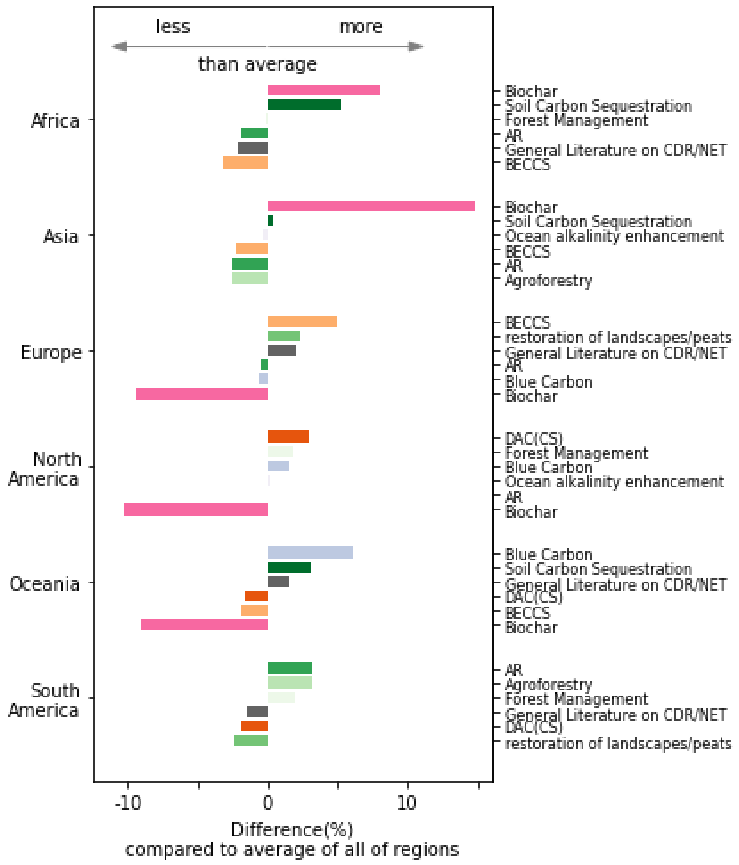


Supplementary Figure 4: Differences of shares in each world region compared to the average of the complete dataset. Colorblind-friendly version of Figure 3 c)

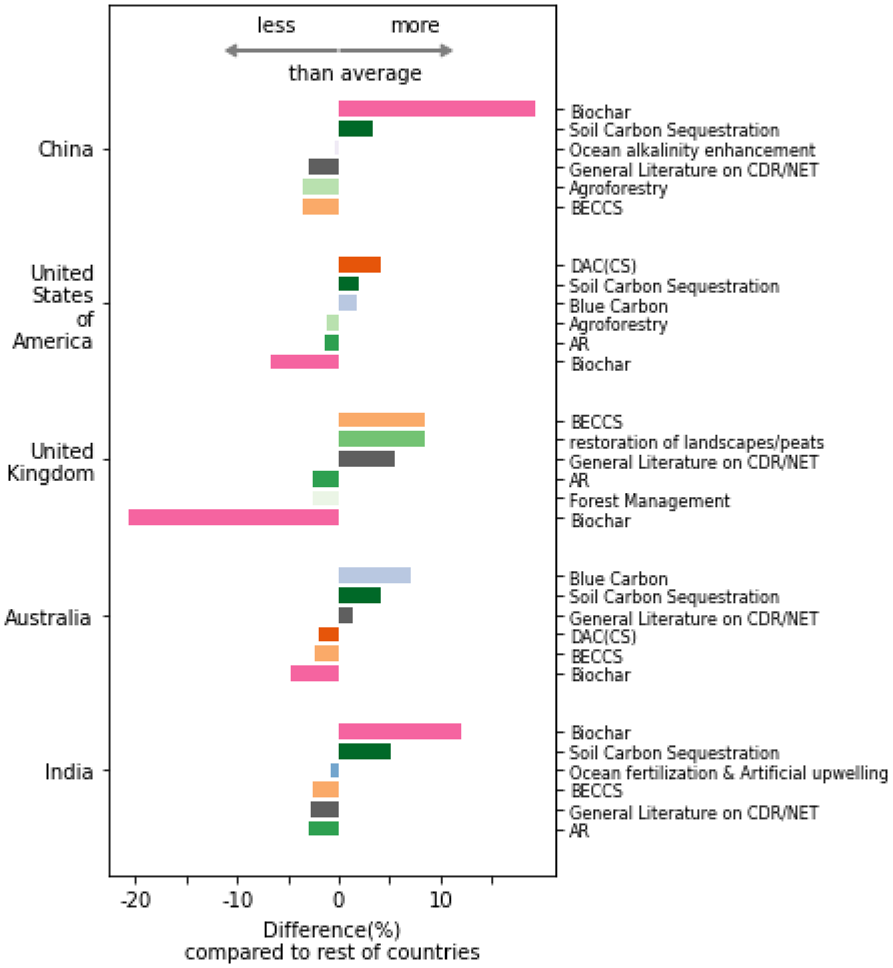


Supplementary Figure 5*:* Related to Figure 4. We compare the percentage difference of the investigated technologies against all others from the complete dataset for the top 5 countries with highest publication count based on first author affiliation. Displayed are only the three highest and the three lowest differences.

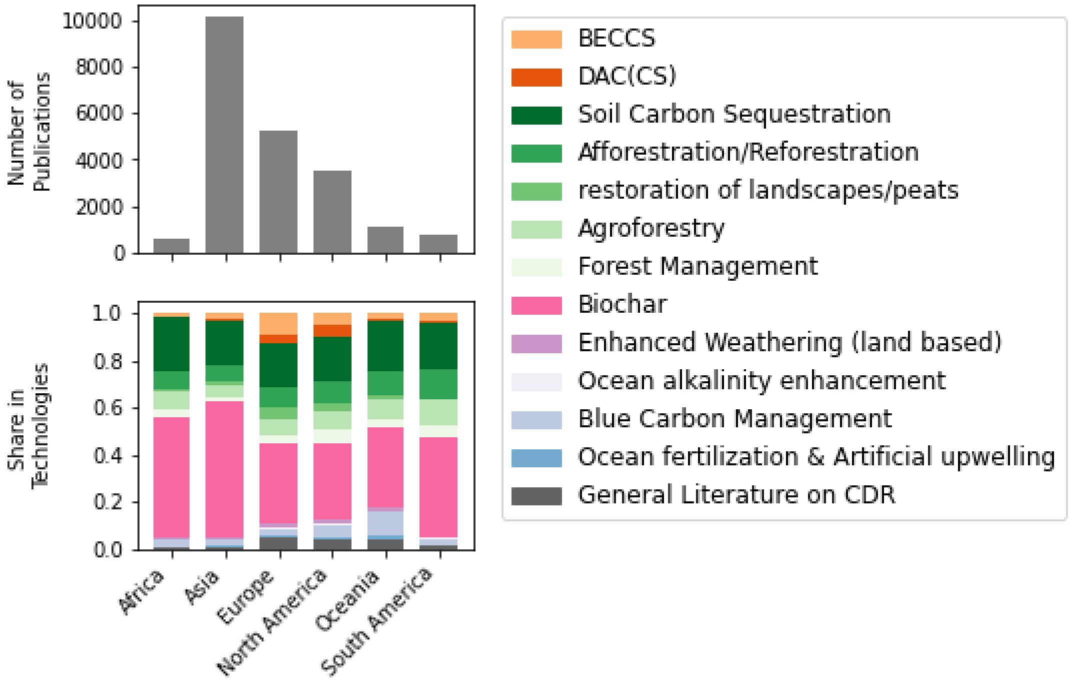


Supplementary Figure 6: Related to Figure 4. Origin of studies derived from the first author affiliation. Shown is the absolute number of studies per world region and the share of CDR technologies per region.

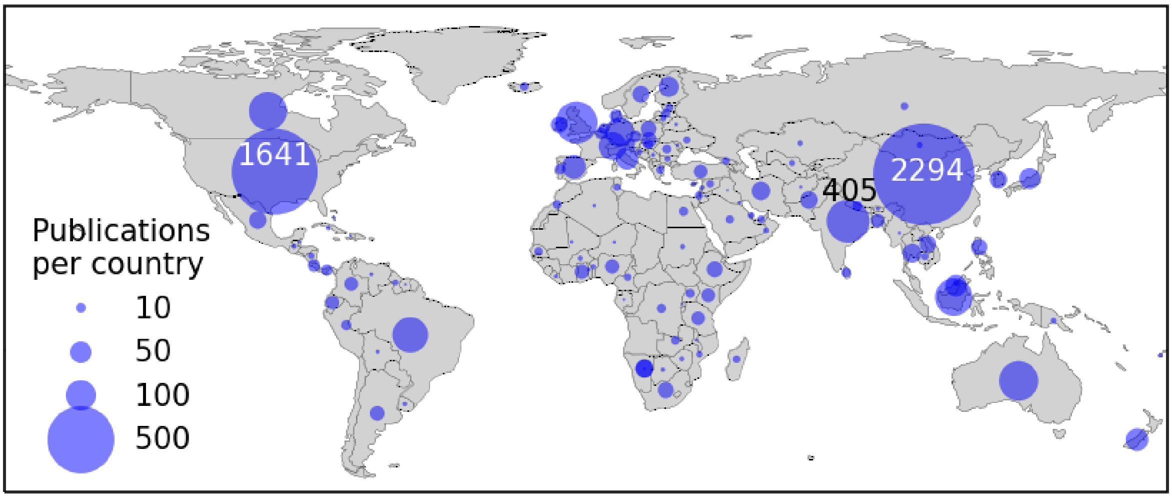


Supplementary Figure 7**:** Related to Figure 4. Number of studies mentioning a location in title/abstract per country based on first author affiliation. The three highest study counts are added.

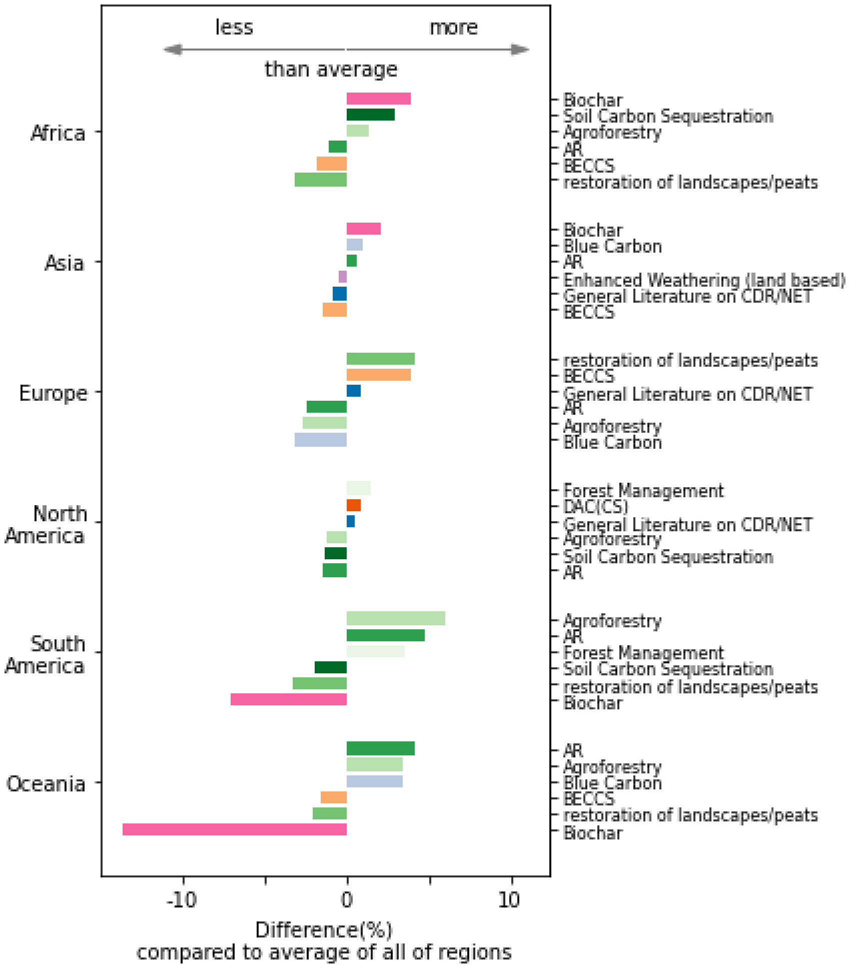


Supplementary Figure 8**:** Related to Figure 4. Similar display as 3b) and Supplementary Figure 5, but only with studies mentioning location in title/abstract: We compare the percentage difference of the investigated technologies against all others from the studies mentioning a location in title/abstract for the world regions countries. Displayed are only the three highest and the three lowest differences.

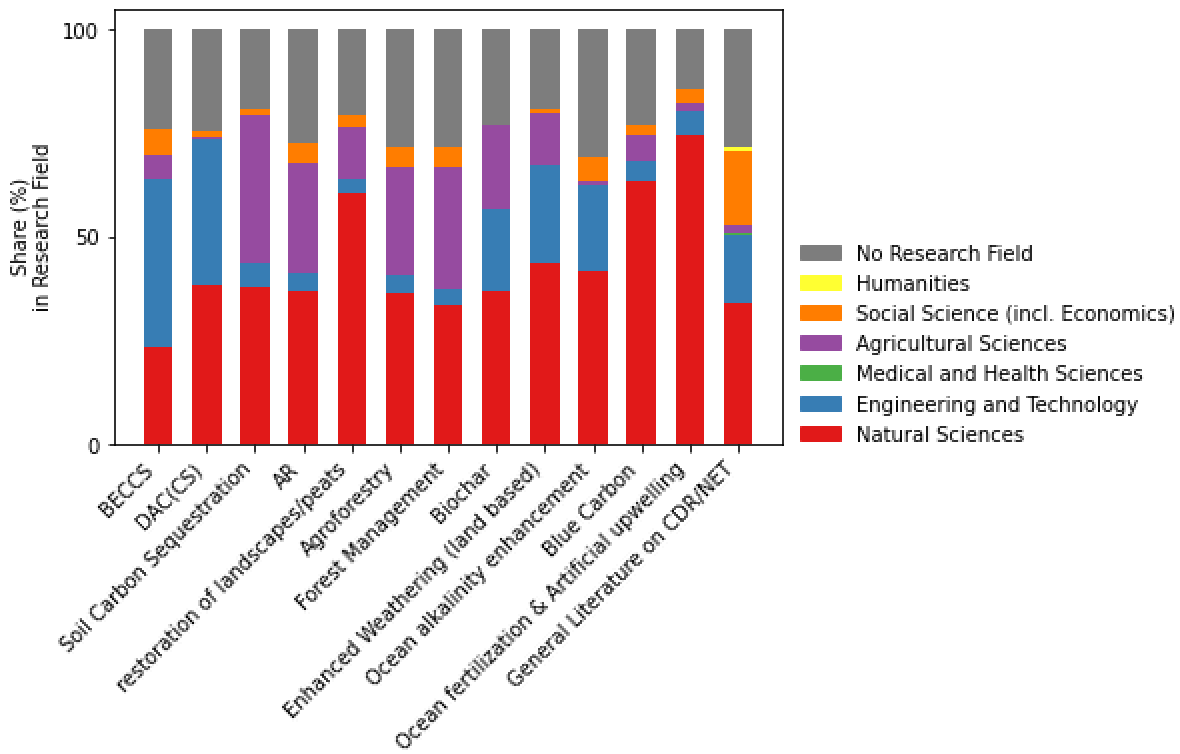


Supplementary Figure 9*:* Related to Figure 5. Share of research fields for each predicted technology together with the documents where the research field is missing due to missing meta-data.

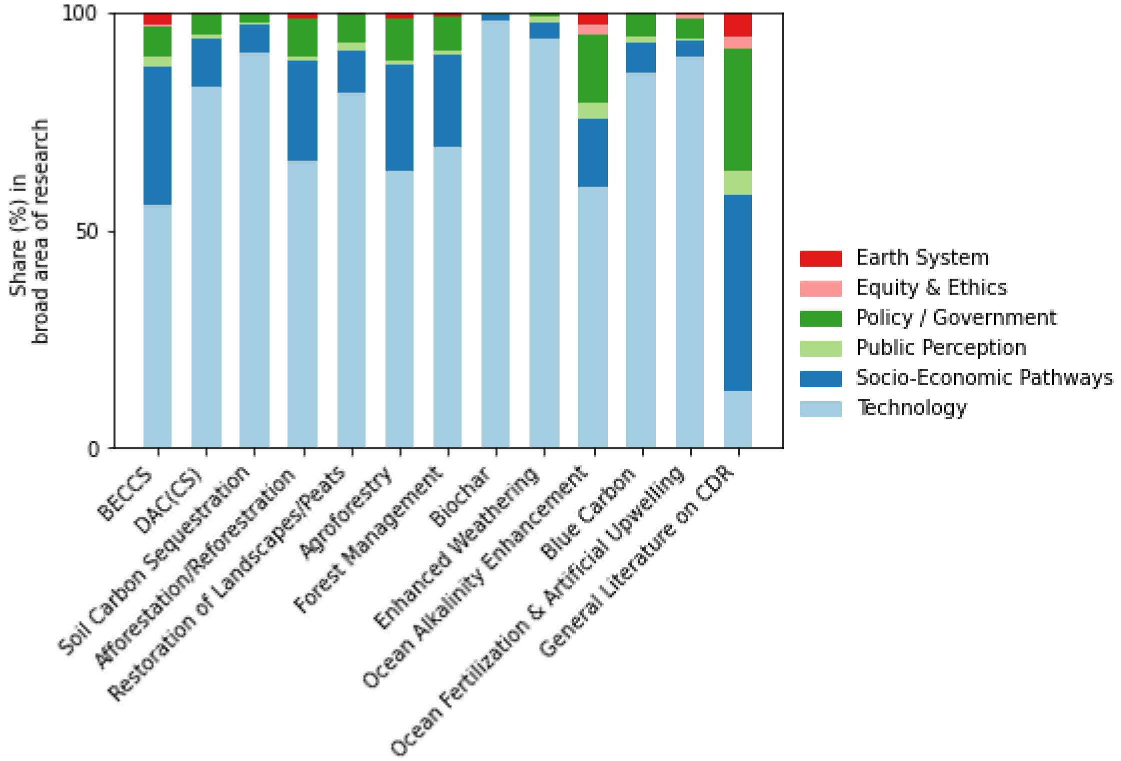


Supplementary Figure 10: Related to Figure 5. Most studies focus on investigating the CDR option from a technical perspective where the technology or ecosystem management method itself is investigated. Share of predicted broad area of research for each technology.

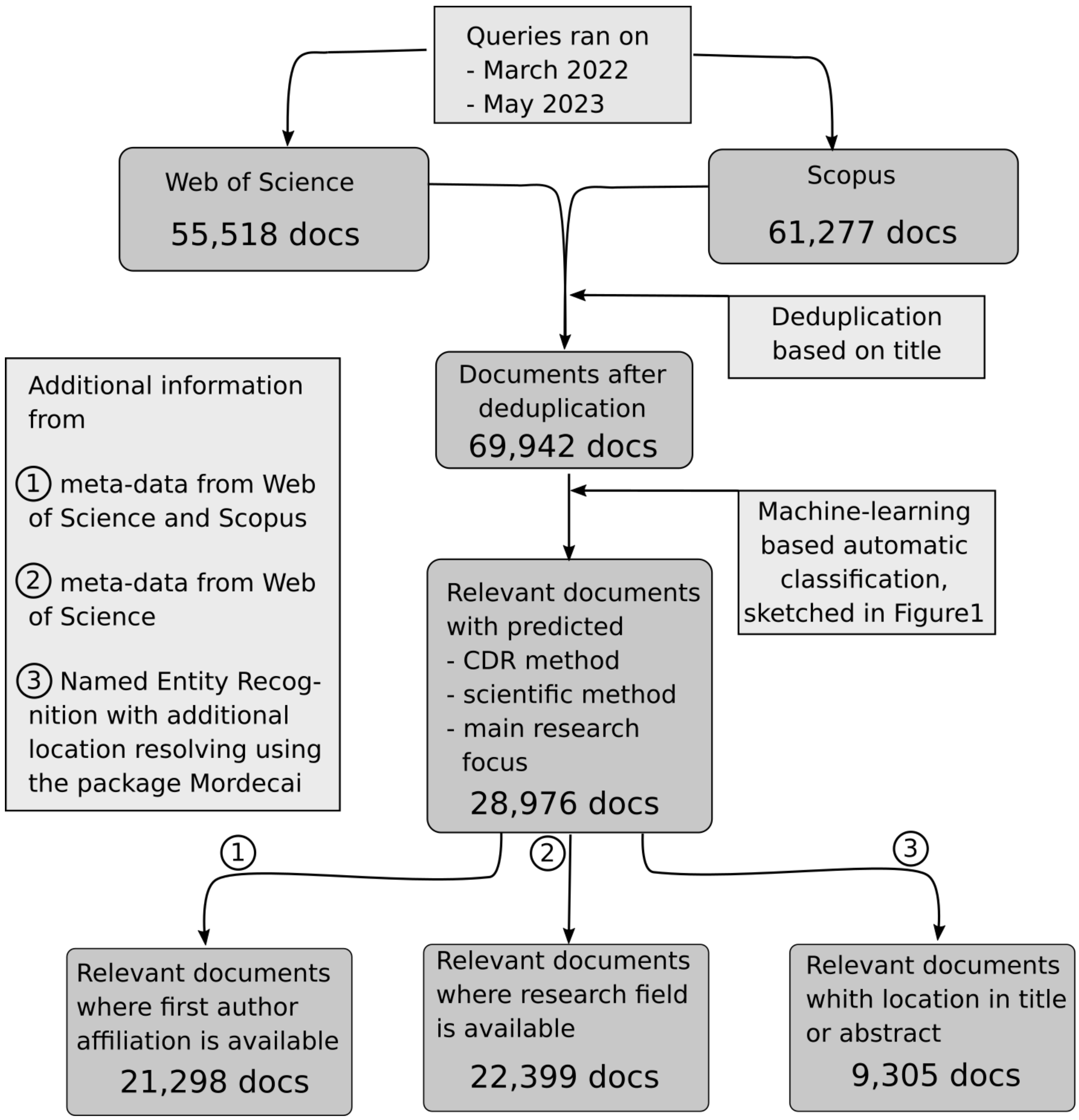


Supplementary Figure 11: Overview of data retrieval together with the number of considered publications in each step. When a share is reported the total number of this step is the basis, e.g. “6,452 studies (30% of all studies where author affiliation is available)” refers to 30% of 21,298 documents.

## Supplementary Tables

Supplementary Table 1: Key numbers for each CDR technology. Numbers of studies and percentages do not add up to the total number of studies (28,976) and 100% since one study can address several technologies. Growth rate per year is only calculated if we find more than 50 studies per year. Average growth rate is only displayed if we calculated the growth rate for the full time range.

| **CDR technology** | **number of studies** | **percentage of studies** | **average growth between 2012 and 2017** | **average growth of the past 5 years** | **average growth of the past 10 years** |
|---|---|---|---|---|---|
| General Literature on CDR/NET | 1,092 | 3.77 | | 0.18 | |
| Afforestation/Reforestation | 3,468 | 11.97 | 0.04 | 0.14 | 0.09 |
| Agroforestry | 2,817 | 9.72 | 0.03 | 0.15 | 0.09 |
| BECCS | 1,617 | 5.58 | 0.12 | 0.12 | 0.12 |
| Biochar | 16,232 | 56.02 | 0.23 | 0.18 | 0.21 |
| Blue Carbon | 1,378 | 4.76 | | 0.22 | |
| DACCS | 822 | 2.84 | | | |
| Enhanced Weathering (land based) | 406 | 1.40 | | | |
| Forest Management | 1,366 | 4.71 | 0.02 | 0.15 | 0.08 |
| Ocean Alkalinity Enhancement | 152 | 0.52 | | | |
| Ocean Fertilisation & Artificial Upwelling | 410 | 1.41 | | | |
| Soil Carbon Sequestration | 6,979 | 24.09 | 0.13 | 0.13 | 0.13 |
| Restoration of Landscapes/Peats | 1,037 | 3.58 | | 0.20 | |
| All Studies on CDR | 28,976 | 100.00 | 0.15 | 0.17 | 0.16 |

Supplementary Table 2: For each CDR option the number of examples in the validation dataset which was used to construct the search queries.

| CDR method | number of examples in the validation dataset |
|---|---|
| BECCS | 32 |
| Biochar | 30 |
| Soil Carbon Sequestration | 27 |
| General Literature on CDR/NET | 25 |
| Ocean alkalinity enhancement | 24 |
| Afforestation/Reforestation | 21 |
| Ocean fertilization & Artificial upwelling | 20 |
| Enhanced Weathering (land based) | 19 |
| DAC(CS) | 13 |
| Blue Carbon | 7 |
| Restoration of landscapes/Peats | 5 |

Supplementary Table 3: Queries used to retrieve the documents from.

| | **query - Web of Science** |
|---|---|
| **General CDR** | (TS = ("Carbon dioxide removal" OR ("CDR" AND ( CO2 OR carbon* )) OR "CO2 removal" OR "greenhouse gas removal" OR "CO2 recovery" OR "carbon recovery" OR "negative carbon dioxide emission*" OR "negative CO2 emission*" OR "negative GHG emission*" OR "negative greenhouse gas emission*" OR "carbonnegative emission*" OR ("negative emission*" AND carbon) OR ("negative emission*"AND CO2)) OR TS = ( geoengineering AND ((carbon OR CO2) NEAR/2 (sequest* OR accumulat* OR storage OR capture))) OR TS= (("ocean solutions" OR "natural climate solutions") AND ("CO2" OR "carbon*")) OR AB= ((("carbon" OR "CO2" OR "carbon dioxide") NEAR/2(sequest* OR remov* or stor*) NEAR/2 "technolog*")) OR TI= ((("carbon" OR "CO2" OR "carbon dioxide") NEAR/2(sequest* OR remov* or stor*) NEAR/2 "technolog*") )) |

| | |
|---|---|
| | NOT TS = ("extracorporeal" or "syngas" or "biogas upgrading") |
| **AR** | TS = ((afforestation OR reforestation)AND("carbon dioxide removal" OR seques* OR storage OR CO2 OR carbon)) |
| **BECCS** | TS = (BECCS OR ((biomass OR bioenerg*) AND ("CCS" OR "Carbon capture and storage" OR "Carbon dioxide capture and storage" OR "CO2 capture and storage"))) |
| **BIOCHAR** | AB = (((biochar* OR bio-char) AND (seques* OR stor* OR carbon OR soil OR agricult* OR agroforestry OR fertil* OR pyrolys*) OR (char AND carbon AND(seques* OR stor* OR sink )))) |
| **Restoration of landscapes/peats** | TS = ("natural climate solution*" AND (("CO2" or carbon) NEAR/3 (remov* OR stor* OR sequest*) OR (restoration AND ("CO2" OR carbon)))) OR<br>TS= (("peat" OR "peatland") NEAR/2 (restor* or manage*) AND ( "CO2" or carbon) NEAR/3 (remov* OR stor* OR sequest* OR "sink") ) OR<br>AB= (("peat" OR "peatland") NEAR/2 (restor*)) |
| **CCS** | TS= ("Carbon capture and Storage" OR "Carbon dioxide capture and Storage" OR "CO2 capture and storage" OR ("Carbon dioxide" OR CO2) NEAR/3("capture and sequestration"OR "recovery and sequestration") OR ((("CO2" OR "carbon") NEAR/3 (captur* OR stor* OR recover* OR extrac* ) )AND ("post combustion" OR scrub*))) |
| **CCUS** | (AB = (("Carbon capture and Storage" OR "CO2 capture and storage" OR "carbon capture and sequestration" OR "CO2 capture and sequestration") NEAR/3 ("Utili*ation" OR "usage")) OR<br>AB = (((CO2 OR carbon ) NEAR/2 "Utili*ation")AND ((CO2 OR carbon ) NEAR/2 (sequestr* OR remov* OR stor* ))))<br>NOT TS= (syngas or EOR or EGR) |
| **Algae farming** | TS = (("*algae" OR "algal" OR "seaweed") AND (( "harves*" OR (("CO2" OR"carbon" )AND ("seques*" OR "stor*"))) )) |
| **Blue carbon** | AB=((((seagrass OR mangrove* OR coastal OR saltmarsh* ) NEAR/3 (afforest* OR rehabilit* OR restor* OR manage*) )OR "blue carbon") AND ((carbon OR CO2 or C) NEAR/5 (sequest* OR accumulat* OR storage OR capture OR burial) or "C t/yr(-1)" )) OR TS=(("seagrass" OR "mangrove*" )NEAR/2"restor*") |
| **DACCS** | TS = (((captur* OR extract) AND (direct* NEAR/3 (air OR atmosph*))) AND (CO2 OR carbon)) OR TS = ((*sorbent OR amine OR membrane) AND capture NEAR/2 (carbon OR CO2) AND ("ambient air" OR "atmospher*")) OR<br><br>AB = ((reduc* OR captur* OR stor* OR extract* OR remov* )NEAR/2 ("CO2" OR "carbon") NEAR/2 ("ambient air" OR "direct air"))<br><br>NOT TS = (phenolic OR PCB* OR particulate OR NOx OR |

| | |
|---|---|
| | isotope OR "heat pump" OR polycyclic OR *bacteria* OR lignin OR sink OR pollution OR biofuel* OR sugar) |
| EW | ((TS= ((geoengineer*) AND (silicate OR olivine OR albite OR CACO3 OR liming)) OR<br>TS= ((silicate OR olivine OR albite OR CACO3 OR liming OR basalt*) AND ((("soil*" OR "cropland" ) AND ("CO2" AND (stor* OR sequestr* OR remov*))) OR (mitigat* NEAR/3 ("climate change" OR "global warming" OR geoengineer*)))) OR<br>TS= ((silicate OR olivine OR albite OR CACO3 OR liming OR basalt*OR mineral OR apatite* OR feldspar* ) AND (agricultur* OR soil) AND (crush* NEAR/5 rock*)) OR<br>TS= ((("enhance*" OR "artificial*") NEAR/2 "weathering" ) AND ((carbon OR CO2 OR "climate change" OR "global warming") NEAR/3 (remov* OR sequest* OR storage OR sink OR mitigat* OR reduc*))) OR<br><br>(TS = (("enhance*" OR "artificial*" OR "chemical" OR "accelerate*" OR "mineral") NEAR/2 "weathering" ) AND TS = ((carbon OR CO2 OR "climate change" OR "global warming") NEAR/3 (remov* OR sequest* OR storage OR sink OR mitigat* OR reduc*) )) AND<br>TS = (mineral* OR rock OR silicate) ) OR<br><br>TS=((enhance* OR accelerate*)NEAR/4 ("carbon dioxide mineralization" OR "CO2 mineralization") AND (weathering)))<br><br>NOT TS = (glaci* OR ice* OR ordovic* OR Aptian OR Cenozo* OR Paleo* OR Mezoso*) |
| OAE | (TS = ((("ocean") AND (alkalin* OR lime*))AND (remov* OR stor* OR sequestr*) AND (CO2 OR carbon*)) OR TS = (("ocean alkalini*") AND (remov* OR storage OR mitigat* OR sequest*) AND (CO2 OR carbon*)) OR TS =( "revers*" NEAR/2 "Ocean acidification" ) OR TS=((("enhanced" OR "artificial" OR "carbonate") NEAR/2 (weathering )) NEAR/2 (coast* OR ocean OR sea) ) )<br><br>NOT TS = (glaci* OR ordovic* OR Aptian OR Cenozo* OR Mezoso* or paleo* OR palaeo* OR "sediment*" ) |
| OF / AU | (TS = (ocean NEAR/5 (fertili*ation OR enrichment) AND iron NOT natural NOT ice* NOT glaci* ) OR<br>TS = ((Pacific OR Arctic OR Indian OR Atlantic ) AND iron NEAR/5 (fertili*ation OR enrichment)<br>NOT natural NOT ice* NOT glaci* ) OR<br><br>TS = ((ocean NEAR/2 "pip*" AND vertical) NOT mining*) OR<br>TS = (ocean NEAR/2 ("upwelling" OR "up-welling")AND artificial )<br>OR TS = (carbon OR CO2) AND TS= ("ocean fertili*ation" OR "enhanced upwelling" NOT glaci* NOT ice* NOT natural ))<br><br>NOT TS=(volcanic or volcano) |

| | |
|---|---|
| **SCS** | TS = (("soil carbon management" OR "soil carbon sequestration") OR ("soil organic carbon" OR (soil NEAR/3 (carbon OR CO2) NEAR/3 (sequest* OR storage OR remov*))) AND (biochar or bio-char)) OR<br>TS = ((soil NEAR/3 (carbon OR CO2) NEAR/3 (sequest* OR storage OR remov*)) AND ("climate change" OR "global warm*") AND (manag* OR practice* OR restoration OR land-use))OR<br>TS=(("carbon farming" OR ("land management" AND (carbon OR CO2) NEAR/3 (sequest* OR storage OR remov*)))) |

Supplementary Table 4: Mean F1-scores and standard deviations in brackets for different classification approaches resulting from a 3-fold validation approach. For multi-label classifiers we use macro averages of the F1 scores. We also present the performance of the final classifier used in the paper, where we performed hyperparameter tuning and adjusted the decision boundary to maximize F1-scores.

| **Classifier** | **F1 score - ClimateBert** | **F1 score - ClimateBert with adjusted threshold** | **F1 score - DistilBert** | **F1 score - SGD + Huber-loss** |
|---|---|---|---|---|
| Relevance (binary) | 0.91 (0.008) | 0.92 (0.008) | 0.90 (0.007) | 0.88 (0.007) |
| Technology (multi-label) | 0.71 (0.086) | 0.76 (0.074) | 0.53 (0.009) | 0.67 (0.006) |
| Method (multi-label) | 0.64 (0.112) | 0.67 (0.128) | 0.38 (0.016) | 0.49 (0.043) |
| Main Focus (multi-label) | 0.71 (0.085) | 0.76 (0.074) | 0.52 (0.009) | 0.69 (0.006) |

Supplementary Table 5: Number of samples in the complete training dataset and mean F1-scores and standard deviations for the different CDR method classes resulting from a 3-fold validation approach.

| **Technology** | **Number of Samples** | **F1 - mean** | **F1 - std** | **ROC - mean** | **ROC - std** |
|---|---|---|---|---|---|
| Afforestation/Reforestation | 107 | 0.65 | 0.16 | 0.78 | 0.11 |
| Agroforestry | 20 | 0.37 | 0.21 | 0.74 | 0.13 |
| BECCS | 351 | 0.92 | 0.05 | 0.97 | 0.01 |
| Biochar | 634 | 0.99 | 0.00 | 0.99 | 0.00 |
| Blue Carbon | 351 | 0.98 | 0.01 | 1.00 | 0.00 |
| DAC(CS) | 246 | 0.89 | 0.06 | 0.93 | 0.06 |
| Enhanced Weathering (land based) | 172 | 0.84 | 0.08 | 0.92 | 0.05 |
| Forest Management | 47 | 0.70 | 0.17 | 0.86 | 0.14 |
| General Literature on CDR/NET | 116 | 0.72 | 0.12 | 0.84 | 0.05 |
| Ocean Alkalinity Enhancement | 50 | 0.70 | 0.17 | 0.83 | 0.16 |
| Ocean Fertilisation & Artificial Upwelling | 95 | 0.89 | 0.09 | 0.93 | 0.06 |
| Soil Carbon Sequestration | 508 | 0.89 | 0.06 | 0.93 | 0.04 |
| Restoration of Landscapes/Peats | 165 | 0.94 | 0.05 | 0.97 | 0.03 |

Supplementary Table 6: Number of samples in the complete training dataset and mean F1-scores and standard deviations for the different scientific method classes resulting from a 3-fold validation approach.

| **Method** | **Number of Samples** | **F1 - mean** | **F1 - std** | **ROC - mean** | **ROC - std** |
|---|---|---|---|---|---|
| Data Analysis / Statistical Analysis / Econometrics | 349 | 0.71 | 0.13 | 0.83 | 0.07 |
| Experimental - Field study | 659 | 0.93 | 0.06 | 0.96 | 0.03 |
| Experimental - Laboratory | 1236 | 0.95 | 0.05 | 0.97 | 0.04 |
| Life Cycle Assessments | 75 | 0.86 | 0.18 | 0.93 | 0.10 |
| Modelling | 747 | 0.92 | 0.06 | 0.96 | 0.03 |
| Qualitative Research | 85 | 0.69 | 0.22 | 0.82 | 0.12 |
| Review | 699 | 0.86 | 0.07 | 0.90 | 0.05 |
| Survey | 37 | 0.64 | 0.18 | 0.82 | 0.12 |
| Systematic Review | 27 | 0.45 | 0.41 | 0.77 | 0.24 |
| Unknown Method | 66 | 0.41 | 0.39 | 0.72 | 0.22 |

# Supplementary Methods

## Additional Information for Data Retrieval

Retrieval of the data can be described in 3 Steps:

We retrieved data from Web of Science and Scopus using queries. The queries were validated with a validation dataset (see Supplementary Method 1).

About 1/10 of all documents were sorted into different categories by humans, the documents were “annotated” (see Supplementary Method 2 and Supplementary Note 1).

These annotations were used to train classifiers - Machine Learning algorithms (Supplementary Method 3 and 4).

We further used additional information to enrich the data as indicated in the corresponding sections.

## Supplementary Method 1: Queries and Validation Dataset

To generate the validation dataset we gathered all 133 citations of the IPCC AR6 WgI Chapter 5.6 and Wg3 Chapter 12.3 [1] and reviewed for each paper title and abstract if it is relevant for the CDR map. 66 out of 133 were then selected. Additionally we took a random sample of 50 papers from the CDR bibliography [2] published by the Climate Protection and Restoration Initiative. The final validation dataset is made available. An overview is presented in Supplementary Table 2.

We then designed queries for each CDR method by collecting keywords. Those queries were then checked if they returned all of the documents of the validation dataset. The queries are shown in Supplementary Table 3.

## Supplementary Method 2: Coding

5,339 documents – 100-600 per CDR method - were checked manually if they are relevant to be included into the map using the NACSOS platform [3]. If they were deemed to be relevant additional labels such as CDR method, scientific method and main focus were added. Each document was coded by two people. Disagreements in codings were discussed and a common label was found among the coders. If the two coders did find a common ground the topic was discussed in a larger round. To stay consistent over the whole coding period we set-up and maintained a coding guideline which contained rules on how to label specific cases [4].

## Supplementary Method 3: Classification

The coding was used to train classifiers in order to extend the labels to the unseen documents.

In the following tables we report on the evaluation metrics for each of the classifiers, the binary classifier to decide for relevance as well as all multi-label classifiers for the different study characteristics. Each score is the result of a 3-fold train/test split where two thirds of the data is used to train and calibrate the classifier and one third is used to evaluate it. This procedure is done 3 times by using each time a different non-overlapping part of the data for testing. In this way we get a good estimate of how a classifier performs on the overall dataset (cf. [5]). For evaluation metrics, we used the F1 score, which is the harmonic mean of precision—indicating the proportion of correctly classified samples within a given class—and recall, which measures the proportion of relevant samples from the validation dataset that were correctly identified. The range of F1 scores goes from 0 to 1 where 1 signifies a perfect classifier. Another reported metric is the ROC-AUC, or Area Under the Receiver Operating Characteristic Curve. This metric orders classified samples by their likelihood of belonging to a specific class, assessing the model's ability to differentiate between classes across various thresholds. A ROC-AUC of 0.5 indicates random performance, while a score of 1 signifies a perfect model.

All final classifiers were then trained on the complete dataset to make use of all information in the labeled dataset.

To find the optimal classification algorithm we tested three different main models: ClimateBert, ClimateBert's origin DisitilBert and tf-idf-encoding together with an SDGClassifier with Huber-loss. We report on the mean F1 scores for each approach in Supplementary Table 4.

Once decided for the main classifier architecture, ClimateBert for every classification task, we used RayTune [6] to decide for the optimal hyperparameters. We investigated learning rate, weight decay, warmup steps, number of epochs and training batch size. Again, we use a 3-fold train/test split to get an estimation of the performance for each classifier. For every train/test split we estimate hyperparameters separately and report on the mean F1 scores on the test datasets, cf. Supplementary Table 5-6.

For the final classifiers used in the publication we estimate the hyperparameters on the complete training dataset.

## Supplementary Method 4: Estimation of Confidence Interval for Absolute Counts

To estimate the confidence interval for the absolute publication count, we adjust the count using the True Positive Rate (TPR) and False Positive Rate (FPR) as shown in

$$p_{adjust} = \frac{p - FPR}{TPR - FPR} \quad (1)$$

$$p = \frac{N_{rel}}{N} \quad (2)$$

$$N_{adjust} = p_{adjust} N \quad (3)$$

(as stated in [7]). Here, N_rel is the number of relevant documents found by the relevance classifier, N is the number of all documents in the complete dataset, FPR and TPR are estimated from the validation procedure described earlier. Since TPR and FPR are proportions, we calculate their confidence intervals using binomial proportion confidence

intervals. The confidence interval for the absolute counts is then obtained by substituting the confidence intervals of TPR and FPR into Eq (1) and Eq (3).

# Supplementary Note 1: Coding Guidelines

In the following we publish all rules which were used to label the documents. The complete coding guideline which includes additionally instructions how to use the NACSOS platform are published in the complete coding protocol [4]

# Inclusion and Exclusion

The first choice for all documents is whether it is relevant or not. In essence, a document is relevant if it discusses Carbon Dioxide Removal (CDR) Technology in any way. This includes discussing the application of a CDR - Technology, its side-effects or societal implications, such as governance or public perception. Note that the technology is not always mentioned explicitly, sometimes rather a process is described that amounts to removing $CO_2$ from the atmosphere and storing it, e.g. Pyrolysis of Biomass resulting in the sequestration of $CO_2$. Long-term storage (*at least 5 years* is important in this context. We are not interested in technology pathways that re-release the carbon after short periods of time. Within this definition, $CO_2$ from direct air capture used in sparkling water or used for the production of synthetic car fuels are not considered as CDR. Although some of the technologies are not regarded as CDR, such as CCS, algae farming or CCUS we include them anyway. We might want to analyse them later..

More detailed inclusion/exclusion criteria are given in the table below. If a document meets all of the inclusion criteria, you click “Yes” on the question for relevance . If it does not meet one or more of the below criteria, click “No” to move to the next document. If you are unsure, click “Maybe”. Articles rated as “Maybe” will be discussed with your colleagues. (Note again that the assignment of relevance should be the last step in coding the record as it immediately saves changes and presents the next record.)

Supplementary Table 7: Inclusion and exclusion criteria for documents during coding

| **Inclusion** | **Exclusion** |
|---|---|
| Any study where $CO_2$ Storage by active and conscious human intervention is discussed and is (potentially) accomplished over a longer period (more than 1 year). This includes any direct discussion of CDR Technologies (explicitly and implicitly) **or** of concrete implications for the application of CDR Technologies. Furthermore, it includes any process of C-fixation, where the end-use is framed in the context of CCS **or** remains open.<br><br>Edge cases to include:<br><br>- biochar is produced but it is not clear what happens to the biochar | Explicitly excluded are<br><br>- studies discussing CCS in the production of biofuels and other short-lived products (e.g. cosmetics etc.) where no net-negative emissions are achieved.<br><br>- studies where biochar is burned<br><br>- studies not dealing with a CDR Technology<br><br>- natural processes where there is no human influence or the data can not be used to influence human actions |
| The abstract must be available in English. *(If no abstract is presented see point 2 below)* | The abstract is only available in languages other than English. |

## General Remarks on Coding Conventions

1. We code very inclusively, meaning:
   All records where the abstract mentions a CDR technology are included **unless** it is only mentioned in the context of concluding **remarks for further research, as a rhetorical figure OR** a pathway **only involves short-term $CO_2$ storage**. Furthermore, if we cannot exclude a paper on the abstract base, we include that record.

2. We use the title, abstract and - if available - the author keywords to judge the study. If an abstract is not available, judge from the title if possible. If the information does not suffice for **an inclusion** click “maybe”. And add the note “No abstract”. A title clearly mentioning Carbon Dioxide Removal, a CDR technology or CDR technologies in general is sufficient for inclusion.

3. We code what has been stated in the abstract and can be regarded as primary research and the objective of the study. If the title/abstract deals with more than one technologies/scientific methods all are coded.

4. Since we want to use machine learning methods to judge the remaining articles, please do not use information besides the title/abstract/keywords to come to an inclusion judgement, rather use the “Maybe” label. You should infer as little as possible to come to a judgement.

5. You may and should refer to author keywords.

6. We do not use the Web of Science Keywords for judgements, as they are apparently automatically generated and often misleading.

7. It is not sufficient to include a study if only one vague reference to CDR or a CDR Technology is mentioned. Include the record if multiple references are given.

# Categories

For all relevant articles we further sort them into 4 different categories, namely CDR technology, research method, main focus of study, side-effects. Please click all categories that apply. This can mean **the categories are not mutually exclusive**. We try to fix coding boundaries in the tables below.

It is possible that none of the options in one or multiple of the categories apply. If the information in the abstract itself is not sufficient to decide what option within a category applies, please do not make a selection. But because all the documents are research articles on CDR we expect for each article that it deals with a CDR technology and uses a research method. Those two categories must be filled for each included article. Do not use outside sources or make speculative inferences.

The tables below present all categories, their labels and label descriptions, furthermore the third column on rules/application provides for some cases additional information on how a given label is applied and edge cases are treated.

## CDR Method

Every included study has to be assigned to a technology. Not mentioning a specific technology gets a “General literature on CDR” label, a technology which cannot be assigned to another technology  a “Other/New technology” label.

Supplementary Table 8: Coding guidelines to label documents with the different CDR methods

| **Label** | **Description/Definition** | **Rule / Application** |
|---|---|---|
| Bioenergy with Carbon Capture and Storage (BECCS) | The application of Carbon Dioxide Capture and Storage (CCS) technology to bioenergy conversion processes. | This label **includes carbon negative** Biofuel production<br><br>If BECCS is applied, we do not code CCS separately. Unless BECCS and CCS are also discussed separately.<br><br>BECCS usually refers to Energy or Heat production in a power plant , where the flue-gases are filtered and stored using CCS.<br><br>If co-firing (using Biomass and fossils together) is discussed, we code both CCS and BECCS<br><br>Mentioning bioenergy alone in the context of mitigation does not suffice for an inclusion. |
| Afforestation and reforestation (AR) | Planting of new forests on lands that historically have not contained forests and replanting of forests that have been cleared. Planting forests per se reduces CO2. Studies mentioning AR are therefore included very inclusively. | |
| Agroforestry | *Agroforestry* is a land use management system in which trees or shrubs are grown around or among crops or pastureland. Agroforestry is one of the CDR technologies mentioned in the latest | |

| | | |
|---|---|---|
| | IPCC report and as such it is always included. | |
| Forest management | Every aspect of management which leads to a sustained carbon dioxide removal. Forest management per se does not increase carbon storage, the already existing storage is rather maintained. Because of that the study must refer to carbon sequestration, e.g. soil carbon, biomass in the forest etc,  to be included into the map. | |
| Direct Air Carbon Capture and Storage (DACCS) | Chemical process by which $CO_2$ is captured directly from the ambient air, with subsequent storage. Also known as direct air capture and storage (DACS). | If DACCS is applied, we do not code CCS separately. Unless DACCS and CCS are also discussed separately.<br><br>We also include DAC-only papers here that focus on the process of dissection C from ambient air.<br><br>Included are also research on DACCS indoors although their capacity is small. |
| Enhanced Weathering (terrestrial) | Enhancing the removal of carbon dioxide ($CO_2$) from the atmosphere through dissolution of silicate and carbonate rocks by grinding these minerals to small particles and actively applying them to soils. | Examples of silicate and carbonate rocks are basalt and feldspar and lime and dolostone. Sometimes components of limestone are mentioned as well e.g. CaCo3.<br><br>Liming is  explicitly included.<br><br>We are interested in active human mediated weathering. **Excluded are:**<br><br>Descriptive analyses of natural weathering processes<br><br>- Non active anthropogenic effects:<br>o Analyses of aerosol effects<br><br>If it is **unclear** whether the EW process is applied on land or in the ocean use Enhanced Weathering as default. |

| | | |
|---|---|---|
| | | If both usages (on land and in the ocean) are described use both labels.<br><br>Liming in agriculture seems to have controversially effects on CO2, it might sequester CO2 or increase CO2 emissions. Because we are not sure about the overall outcome we will include papers on liming in agriculture.<br><br>Ocean liming is a one way to in enhance ocean alkalinity and is included as “Ocean alkalinity enhancement”. |
| Ocean alkalinity enhancement (enhanced ocean weathering) | Enhancing the removal of carbon dioxide from the atmosphere through dissolution of silicate and carbonate rocks by grinding these minerals to small particles and actively applying them to coasts and oceans. | Is the opposite of acidification.<br><br>We are interested in active human mediated weathering. **Excluded are:**<br>- Descriptive analyses of natural weathering processes<br>- No active anthropogenic effects:<br>    o Analyses of aerosol effects |
| Ocean fertilization & Artificial upwelling | Deliberate increase of nutrient supply to the near-surface ocean in order to enhance biological production through which additional carbon dioxide from the *a*tmosphere is sequestered. This can be achieved by the addition of micro-nutrients or macro-nutrients.<br><br>Upwelling refers to the use of pipes or other methods to pump nutrient-rich deep ocean water to the surface where it has a fertilizing effect. | |
| Biochar | Stable, carbon-rich material produced by heating *biomass* in an oxygen-limited environment (pyrolysis) . | < (e.g. Sorption etc.) and studies discussing the co-production of biochar and biofuel are **included**.<br><br>We **include** Hydrochar under this label.<br><br>Uses of Biochar as fuel (where it is burned and $CO_2$ released) are |

| | | |
|---|---|---|
| | | **excluded**:<br>- E.g. Direct fuel cell applications |
| Soil Carbon Sequestration (SCS) | Land management changes which increase the soil organic carbon content, resulting in a net removal of $CO_2$ from the atmosphere. | Applications of biochar as soil amendment are included if the effects on the C content of the soil is discussed.<br><br>“crop residue retention” is practice used for SCS<br><br>We **include** records dealing with cover crops only if their application is framed in a CDR context.<br><br>It seems cultivation of biofuel crops is beneficial for long term storage of soil carbon. We include those cases and label them with SCS and bioenergy.<br><br>**Sediment carbon**, i.e. carbon stored in materila which sank to the ground in water, is no soil carbon. We do not code SCS here.<br><br>**Carbon Farming** is a practice where farming serves to sequester carbon in soil. We will code it as SCS but it can include other technologies, such as biochar. |
| Blue carbon | Blue carbon is the carbon captured by living organisms in coastal (e.g., mangroves, salt marshes, seagrass beds, macro algae and seaweed) and marine ecosystems, and stored in biomass and sediments. | We include all discussion of **restoration** efforts of Blue Carbon (often dealing with mangrove forests and seagrass beds), either before restoration or evaluation of already existing restored forests/beds<br><br>Included is **conservation** of marine ecosystems (mangroves etc.) if it is done with a focus on carbon sequestration.<br><br>Coral reefs are not a clear Blue carbon technology as their potential of sequestering carbon is low. If they are discussed with a focus on Blue Carbon we follow the restoration/conservation rules.<br><br>Mentioning “management” without mentioning carbon sequestration or looking at the sequestered carbon will not be included, cp. forest management.<br><br>Blue carbon often manifests in sediment carbon. Sediment consists of material which sank to the ground. It is **no** soil carbon. |

| | | |
|---|---|---|
| Restoration of landscapes and peats | A process by which formerly destroyed landscapes that sequester $CO_2$ are restored to their natural conditions. | Is only applied to land-based activities.<br>Mere rewetting for peats is not sufficient, a record needs to state that CO2 is sequestered.<br><br>Should only be double coded with AR and SCS if either AR or SCS are discussed additionally. |
| Carbon dioxide Capture Utilization and Storage (CCUS) | A process in which $CO_2$ is captured and then used to produce a new product. Only if the $CO_2$ is stored in a product for a climate-relevant time horizon, this is referred to as carbon dioxide capture, utilisation, and storage. Only then, and only combined with $CO_2$ recently removed from the atmosphere, can CCUS lead to carbon dioxide removal. *Examples would be usage for furniture, as building material,* | Included are in general products which are solid, excluded are products which are highly flammable. More detailed inclusion/exclusion list: link<br>Enhanced oil recovery (EOR) is excluded as study and does not count as CCUS. Exceptions are if EOR is discussed as part of BECCS/DACCS etc.<br>We observe that many studies talk about CCUS but actually mean CCS. We code here CCS<br>If several long lived and short lived products are discussed we include the study |
| Other/New Technology | Any technology that removes $CO_2$ from the Atmosphere and is not listed in this overview. | Applied to any new technology that comes up. Be reminded of the wide definition technology employed here. |
| General Literature on CDR and CDR Technologies. | Any literature that discusses the process or impact of CDR-Technologies, without a limited focus on one or multiple explicit technologies. | If a record compares multiple specific CDR Technologies all single labels are applied plus "General literature".<br><br>If a record deals with a class of multiple technologies - e.g. terrestrial or marine CDR – use general literature. Also, if a record deals with a (partly) unknown portfolio of CDR technologies, use the labels for the **known** technologies and "general literature" additionally.<br><br>**Included** are papers that explicitly deal with scenarios excluding CDR Technologies and discussing that exclusion. |
| Direct Ocean Capture | Electrochemical processes for | Since apparently the terminology is not fixed yet and some abstracts use Ocean Alkalinity Enhancement and Direct Ocean Capture synonymously we label for processes using electrochemical processes to capture carbon |

| | | |
|---|---|---|
| | oceanic carbon removal<br><br>Note: Only coded for a few documents as part of an additional labelling for OAE, to be found in NACSOS2 in the project “CDR to find OAE” | in the ocean both labels: OAE and Direct Ocean Capture |

## Scientific Method

Please assign to each included article a scientific method. If you are unsure what method is used in the article use “Unknown method”.

Supplementary Table 9: Coding guidelines to label documents with the different scientific methods

| **Label / Subgroup** | **Description** | **Rule / Application** |
|---|---|---|
| Life Cycle Assessments (LCA) | Analysis of all environmental impacts of a product or service over its lifetime. By quantifying all inputs and outputs of material flows and assessing how these material flows affect the environment. In the context of CDR often assessments of C storage with regard to material flows. | - Is related to both statistical analysis/accounting and modelling<br>- If the LCA label is applied do not double code *statistical analysis* or *modelling* |
| Modelling | (Mathematical) simulation of socio-economic systems, energy systems, technological systems, human processes | We also code modelling if there are concrete quantified predictions of the future mentioned in the abstract.<br>Aspen Plus is a software for simulating industrial processes and an indicator for “modelling”.<br>Other words which indicate modelling:<br>- techno-economic assessment<br>- IAM, integrative assessment modelling |

| | | |
|---|---|---|
| | | - scenario |
| Experiment | The manipulation of variables to establish cause and effect relationships. | For all experiments we do not label data analysis additionally if they do some basic calculations, e.g. mean, stdv etc.<br><br>More complex analysis asks for an additional label in data analysis, |
| *Experiment - Field study* | Testing a hypothesis outside of artificial and highly controlled settings. The idea is to include natural variation into the experimental setting. | Includes observational studies.<br><br>Mesocosm experiment, e.g. a tank in the ocean, are labelled as field study. |
| *Experiment - Laboratory experiments* | Testing a hypothesis in an artificial and highly controlled setting of a laboratory. | Microcosm studies where an ecosystem is simulated are labelled as laboratory experiments.<br><br>Includes pot experiments in greenhouses. |
| Review | Any study that relies on a literature review as scientific basis for its claims. | This is understood very broad in the context of this research and **includes:**<br>- Overview pieces (e.g.in book chapters)<br>- Perspective pieces that rely limited and sometimes skewed literature bases (often these do not provide methods and give an introduction into a topic/ overview of what research needs to be done)<br>- Introductions to journal volumes<br>- Proceedings paper are **no** reviews automatically but preliminary research papers, if possible identify a method of all available methods, including review<br>- commentary are included as reviews |
| Systematic review | A type of evidence synthesis, using repeatable analytical methods to collect secondary data and analyse it (quantitatively and qualitatively). It is often mentioned as method in the | Mentioning numbers of reviewed /synthesised articles in the abstract is an indication for a systematic review.<br><br>Meta-analysis, i.e. the comparison and evaluation of several data sets, can be a part of a systematic review. But if the study only conducts a |

| | | |
|---|---|---|
| | abstract. | meta-analysis we code it as “data analysis” |
| Survey | Using any physical or digital tool to gather answers to a predefined set of questions. | also included here are interviews, e.g. with experts |
| Data analysis / Statistical analysis / Econometric | Data analysis, remote sensing analysis,, statistical models, e.g. panel analysis and accounting studies | Meta-analysis, i.e. the comparison and evaluation of several data sets, can be a part of a systematic review. But if the study only conducts a meta-analysis we code it as “data analysis”.<br>Included here are:<br>- machine learning approaches<br>- multi-criteria analysis<br>- statistical linear-mixed effect models |
| Qualitative research | This label is attributed to any qualitative design not caught by other labels. This might include focus groups, case studies, developing frameworks for assessments etc. | Double check if the article does not qualify as perspective (review category) before applying this label. |
| Unknown method | It is the default label if none of the other labels are applicable. | Double check if the article does not qualify as perspective (review category) before applying this label. |

## Main focus

This category aims at catching the wider context in which a technology or its application are discussed in an article.

Supplementary Table 10: Coding guidelines to label documents with the main foci

| **Label** | **Description** | **Rule / Application** |
|---|---|---|
| Public perception & acceptance | Developments of opinions/stances in a given population and media coverage. | |
| Policy, politics and governance | We follow the understanding of "public policy" in order to be as broad as possible in this category: Public policy is an institutionalised proposal or a decided set of elements like laws, regulations, guidelines, and actions to guide CDR implementation. | |
| Earth system/ Climate Systems | Global ocean-, land- and air-systems or their interactions and respective outcomes with regard to Climate Change. This includes management, intervention through deploying CDR technologies and their estimated outcomes. | This category is **limited to:**<br>Quantitative assessments e.g. in the form of climate (consequences) modelling<br>This label usually co-occurs with modelling as method.<br><br>Hints are usage of following scenario models:<br>MAgPIE, LPJ, LPJmL, JSBACH, the name carbon cycle-climate model (see http://climatemodels.uchicago.edu/) |
| Socio-economic pathways | Societal- and market decisions and trajectories. They elicit options for actions. A classical example would be to investigate with a model deployment strategies for different | This **includes:**<br>- Market modelling<br>- Emission pathways<br>- modelling of scenarios<br>- Energy system portfolios |

| | | |
|---|---|---|
| | technologies under economic constraints. Here, we do not include optimal deployment of one technology, this would be labelled as technology specific. | - Integrated assessments<br>- Government investments<br>- Land-use depending on the context it is discussed<br><br>This label usually co-occurs with modelling as method.<br><br>Not included are optimal solutions for one technology, e.g. best suitable land for planting. 4377360. We code such documents as technology.<br><br>Often these documents are framed in a way that they want to guide policies, e.g. If they just mention policy in an outlook, we do not code the policy label here. |
| Technology | Features, processes, and the application of the technology. | This is the most vague/catch-all label in this category. As many articles revolve around technicalities of a given CDR Technology and do not contextualise further.<br><br>If another context category (e.g. Earth Systems) is applied only code this label if, the record discusses variables of/ variations to the process of the CDR technology additionally. |
| Equity & Ethics | Analysis or discussions of the ethical/ normative dimension of CDR technologies and their application. | This might include, risk assessments, assessments of burden-sharing and the like |

## Supplementary References


1. IPCC. *Climate Change 2022: Mitigation of Climate Change. Contribution of Working Group III to the Sixth Assessment Report of the Intergovernmental Panel on Climate Change*. (Cambridge, UK and New York, NY, USA, 2022).
2. Burns, W. Bibliography: Greenhouse Gas Removal / Negative Emissions Technologies – Climate Protection and Restoration Initiative. (2021).
3. Callaghan, M., Müller-Hansen, F., Hilaire, J. & Lee, Y. T. NACSOS: NLP Assisted Classification, Synthesis and Online Screening. Zenodo https://doi.org/10.5281/zenodo.4121526 (2020).
4. Lück, S. *et al.* A Coding Protocol for Labeling Scientific Literature on Carbon Dioxide Removal to Train Machine Learning ModelsCoding Protocol. dx.doi.org/10.17504/protocols.io.e6nvwqwqwvmk/v1 (2022)
5. Callaghan, M. *et al.* Machine-learning-based evidence and attribution mapping of 100,000 climate impact studies. *Nat. Clim. Change* **11**, 966–972 (2021).
6. Liaw, R. *et al.* Tune: A Research Platform for Distributed Model Selection and Training. Preprint at https://doi.org/10.48550/arXiv.1807.05118 (2018).
7. Moreo, A. & Sebastiani, F. Re-Assessing the 'Classify and Count' Quantification Method. Preprint at https://doi.org/10.48550/arXiv.2011.02552 (2021).
